# A Tripartite Interaction Among the Calcium Channel α_1_- and β-Subunits and F-Actin Increases the Readily Releasable Pool of Vesicles and Its Recovery After Depletion

**DOI:** 10.3389/fncel.2019.00125

**Published:** 2019-05-03

**Authors:** Gustavo A. Guzman, Raul E. Guzman, Nadine Jordan, Patricia Hidalgo

**Affiliations:** ^1^Institute of Complex Systems 4, Zelluläre Biophysik, Forschungszentrum Jülich, Jülich, Germany; ^2^Institute of Biochemistry, Heinrich-Heine University, Düsseldorf, Germany

**Keywords:** calcium channel, Ca_V_β subunits, F-actin, RRP size, RRP refilling, EPSC

## Abstract

Neurotransmitter release is initiated by the influx of Ca^2+^ *via* voltage-gated calcium channels. The accessory β-subunit (Ca_V_β) of these channels shapes synaptic transmission by associating with the pore-forming subunit (Ca_V_α_1_) and up-regulating presynaptic calcium currents. Besides Ca_V_α_1,_ Ca_V_β interacts with several partners including actin filaments (F-actin). These filaments are known to associate with synaptic vesicles (SVs) at the presynaptic terminals and support their translocation within different pools, but the role of Ca_V_β/F-actin association on synaptic transmission has not yet been explored. We here study how Ca_V_β_4_, the major calcium channel β isoform in mamalian brain, modifies synaptic transmission in concert with F-actin in cultured hippocampal neurons. We analyzed the effect of exogenous Ca_V_β_4_ before and after pharmacological disruption of the actin cytoskeleton and dissected calcium channel-dependent and -independent functions by comparing the effects of the wild-type subunit with the one bearing a double mutation that impairs binding to Ca_V_α_1_. We found that exogenously expressed wild-type Ca_V_β_4_ enhances spontaneous and depolarization-evoked excitatory postsynaptic currents (EPSCs) without altering synaptogenesis. Ca_V_β_4_ increases the size of the readily releasable pool (RRP) of SVs at resting conditions and accelerates their recovery after depletion. The enhanced neurotransmitter release induced by Ca_V_β_4_ is abolished upon disruption of the actin cytoskeleton. The Ca_V_α_1_ association-deficient Ca_V_β_4_ mutant associates with actin filaments, but neither alters postsynaptic responses nor the time course of the RRP recovery. Furthermore, this mutant protein preserves the ability to increase the RRP size. These results indicate that the interplay between Ca_V_β_4_ and F-actin also support the recruitment of SVs to the RRP in a Ca_V_α_1_-independent manner. Our studies show an emerging role of Ca_V_β in determining SV maturation toward the priming state and its replenishment after release. We envision that this subunit plays a role in coupling exocytosis to endocytosis during the vesicle cycle.

## Introduction

Calcium entry through Ca_V_2.x high-voltage activated calcium channels is a pivotal step during action potential-evoked neurotransmitter release and synaptic plasticity (Wheeler et al., 1994; Cao and Tsien, 2010; Simms and Zamponi, 2014; Nanou and Catterall, 2018). The Ca_V_2.x calcium channel core complex in the mammalian brain is composed of one Ca_V_α_1_ and one Ca_V_β subunit (Müller et al., 2010).

The Ca_V_β family belongs to the membrane-associated guanylate kinases (MAGUKs) class of scaffolding proteins encompassing two highly conserved domains, a Src 3 homology (SH3) domain and a guanylate kinase (GK) domain that are flanked by variable regions (Chen et al., 2004; Opatowsky et al., 2004; Van Petegem et al., 2004). The four Ca_V_β isoforms described until now, Ca_V_β_1_ to Ca_V_β_4_, associate with a highly conserved sequence among the high-voltage activated Ca_V_α_1_ referred to as α_1_ interaction domain (AID) that is located within the intracellular loop joining the transmembrane domains I and II (Pragnell et al., 1994). Association of Ca_V_β with the AID site increases calcium current densities by altering the biophysical properties of the channel and its expression at the plasma membrane (Buraei and Yang, 2010). Accordingly, in mouse hippocampal neurons exogenously expressed Ca_V_β_4_, the predominant Ca_V_β isoform associated with the Ca_V_2.x core complex (Müller et al., 2010), increases neurotransmitter release *via* slowing down voltage-dependent inactivation and promoting Ca_V_2.x channel cell surface expression (Wittemann et al., 2000; Xie et al., 2007; Etemad et al., 2014).

Several lines of evidence suggest that Ca_V_β_4_ affects synaptic transmission by mechanisms that are independent of the upregulation of Ca_V_2.x-mediated currents. Dissociation from Ca_V_α_1_ induced by membrane depolarization favors the association of Ca_V_β_4b_ with a phosphatase 2A regulatory subunit causing their translocation to the nucleus and regulation of transcriptional activity (Tadmouri et al., 2012; Ronjat et al., 2013). Ca_V_β_4b_ also associates with the Rab3 interacting molecule RIM1 and facilitates synaptic transmission by supporting the docking of synaptic vesicles (SVs; Kiyonaka et al., 2007).

We have previously shown that the Ca_V_β_2_ isoform associates directly with F-actin and facilitates the trafficking of Ca_V_α_1_-containing transport vesicles toward the plasma membrane (Stölting et al., 2015; Conrad et al., 2018). The actin-based cytoskeleton supports readily releasable pool (RRP) recruitment, docking step and recycling of SVs as well as of synaptic proteins after neurotransmitter release (Cingolani and Goda, 2008; Hallermann and Silver, 2013; Tanifuji et al., 2013; Hayashida et al., 2015; Rust and Maritzen, 2015; Miki et al., 2016). These two lines of evidence motivated us to investigate whether the interaction between Ca_V_β and the actin cytoskeleton play a role in the recruitment of SVs to the active zone and replenishment after depletion. In order to dissect Ca_V_α_1_-dependent and independent functions (Hidalgo and Neely, 2007; Hofmann et al., 2015; Rima et al., 2016), we generated a wild-type (WT) Ca_V_β_4b_ and a mutant version with disrupted binding to Ca_V_α_1_ (Opatowsky et al., 2004) and, expressed these constructs in primary mouse hippocampal neurons.

We found that Ca_V_β_4b_ also binds to actin filaments (F-actin) and, at excitatory synapses it enhances spontaneous and evoked postsynaptic currents as well as the size of the RRP of SVs and its recovery time after depletion. The enhanced synaptic transmission relies on the association of Ca_V_β_4b_ with Ca_V_α_1_ and depends on an intact actin cytoskeleton. Ca_V_β_4b_ mutant retains the capability to interact with F-actin and to recruit SVs to the RRP, but it fails to increase neurotransmitter release. Our results add a new function of Ca_V_β_4_ in shaping synaptic transmission and expand the already broad functional repertoire of this subunit.

## Materials and Methods

### cDNA Constructs

For heterologous expression of Ca_V_2.2/Ca_V_β_4b_ in HEK293T cells, the cDNA-encoding region of the human Ca_V_2.2α_1_ pore-forming subunit of voltage-gated calcium channels (UniProtKB: Q00975-1) was subcloned into pEGFP-N1 to yield a C-terminal Ca_V_2.2-GFP fusion protein. The human WT Ca_V_β_4b_ (UniProtKB: O00305.2) was fused to mCherry to facilitate recognition of transfected cells. For recordings in hippocampal neurons, cDNA-encoding WT and Ca_V_α_1_-association deficient Ca_V_β_4b_ mutant where fused to eGFP and subcloned into FsY1.1 G.W lentiviral transfer vector (kindly provided by Dr. M Filippov, Nizhny Novgorod, Russia) to yield fusion constructs with the eGFP moiety fused at the C-terminus of Ca_V_β_4b_. To express a Ca_V_β_4b_ mutant with impaired Ca_V_α_1_-association M238A/L384A amino acid substitutions were introduced by PCR based techniques. For protein expression in *E.coli*, the coding region of the core regions of Ca_V_β_4b_ (residues 50–408) or Ca_V_β_2a_ (UniProtKB: Q8VGC3-2) was subcloned into pRSETB vector (Invitrogen, Carlsbad, CA, USA) as described (Stölting et al., 2015). All constructs were verified by DNA sequencing.

### Recombinant Proteins

Histidine-tagged Ca_V_β derivatives were purified from *E.coli* lysates as previously described (Hidalgo et al., 2006). In brief, proteins were purified from the soluble fraction of the crude lysate by metal affinity followed by size-exclusion chromatography. Fractions containing the purified proteins were concentrated and stored at −80°C until use. The glutathione S-transferase (GST) protein alone or fused to the Ca_V_α_1_-anchoring domain (AID) have been previously described (Miranda-Laferte et al., 2014). The GST pull-down assay was done as previously (Hidalgo et al., 2006) and also described in Supplementary Material.

### F-Actin Cosedimentation Assay

Binding of the Ca_V_β derivatives to F-actin was studied using the F-actin co-sedimentation assay according to the manufacturer’s instructions (Cytoskeleton, Inc, Denver, CO, USA) and as previously described (Stölting et al., 2015). Each assay was performed in a volume of 50 μl. For each reaction, either protein alone (control) or together with F-actin, the same amount and stock of Ca_v_β was used. Briefly, the purified proteins were incubated with rabbit muscle actin in actin polymerization buffer containing (in mM): 10 Tris-HCl, 0.2 CaCl_2_, 50 KCl, 2 MgCl_2_, 1 ATP, pH 8.0, centrifuged for 1 h at 150,000× *g* at 4°C in a Beckman TLA 100.1 rotor. After centrifugation, the whole supernatant was transferred to a new tube while the pellet was resuspended in a final volume of 50 μl containing 1× SDS-loading buffer. Supernatant and pellet fractions were then resolved by denaturing SDS-PAGE. Each lane was loaded with 25 μl to permit direct comparison of protein amounts bound or unbound to F-actin. Since Ca_V_β_4b_ core and actin exhibit overlapping migration in the mini gels (Bio-Rad, Hercules, CA, USA), the proteins were resolved using a Multigel-Long chamber (Biometra, Göttingen, Germany). Proteins were visualized with Coomassie Blue and the prestained protein markers Dual color (Bio-Rad, Hercules, CA, USA) or PageRuler Plus (Thermo Fisher, Waltham, MA, USA) were used as molecular mass standards. All assays were repeated at least three times.

### Cell Culture and Immunocytochemistry

Autaptic and mass cultures of hippocampal neurons were prepared from postnatal day 0 to day 3 C57/BL6-N mice and maintained as previously described (Guzman et al., 2010). Briefly, hippocampi were isolated from brain and enzymatically treated with 15 units of papain (Worthington Biochemical Corp, Lakewood, NJ, USA) for 20 min at 37°C. After enzymatic digestion, neurons were mechanically dissociated. For autaptic cultures, neurons were diluted to a density of 1,000 cells/ml and plated onto micro-islands containing glial cells that were cultured 3–5 days prior to seeding neurons. For mass cultures, isolated neurons were diluted to a density of 300 cells/cm^2^ on 25 mm glass coverslips previously treated with poly-D-lysine (Sigma-Aldrich, St. Louis, MO, USA). Neuronal cells were grown in NBA (Invitrogen, Carlsbad, CA, USA), supplemented with Glutamax at 1% (Thermo Fisher, Waltham, MA, USA) penicillin/streptomycin at 2% (Thermo Fisher, Waltham, MA, USA) and B-27 at 2% (Thermo Fisher, Waltham, MA, USA). Neurons were used after 10–14 days *in vitro* (DIV) for electrophysiological recordings and for fluorescence confocal microscopy imaging. HEK293T cells (Sigma-Aldrich, St. Louis, MO, USA) used for electrophysiological recordings of heterologously expressed Ca_V_2.2 were cultivated in cell culture dishes using DMEM (Gibco) medium supplemented with 10% FBS and with 2% penicillin/streptomycin. All cells were cultivated at 37°C and in a humidified atmosphere with 5% CO_2_.

### Virus Production, Transduction of Hippocampal Neurons and HEK Cells Transfection

To deliver the cDNA encoding for the Ca_V_β_4b_ constructs hippocampal cultures were infected with lentivirus. The helper plasmids pRSVREV, pMDLg/pRRE, and vesicular stomatitis virus G protein expressing plasmid were kindly provided by Dr. Thomas Südhof (Howard Hughes Medical Institute, Stanford University, Stanford, CA, USA). Lentivirus was produced (Barde et al., 2010) and transduced as previously described (Stölting et al., 2015). Calcium phosphate transfection was used to co-transfect the lentiviral transfer vector and the three helper plasmids into HEK293FT cells. After 14 h transfection, cell culture medium containing (DMEM, 10% FBS, 100 mM sodium pyruvate, 100 mM non-essential amino acids, and 100 mM Glutamax) was renewed. Solutions were purchased from Thermo Fisher (Waltham, MA, USA). Cell culture medium containing lentiviral particles was withdrawn from the cell surface and ultracentrifuged for 2 h. Lentiviral particles were immediately resuspended in culture medium, frozen in liquid nitrogen and stored at −80°C. Hippocampal neurons were infected using 30–50 μl of viral suspension at 1–3 DIV.

HEK cells were transiently co-transfected with Ca_V_2.2-GFP and either with WT or mutant Ca_V_β_4b_-mCherry using Lipofectamine 2000™ (Invitrogen, Carlsbad, CA, USA). Cells were split 12 h after transfection, and the electrophysiological experiments were performed 24 h later.

### Immunostaining and Confocal Microscopy

Mass hippocampal neurons infected with lentiviral particles encompassing the Ca_V_β_4b_-eGFP constructs were fixed with 4% paraformaldehyde (PFA) in PBS during 10 min at room temperature (RT). PFA was removed and 0.1% Triton X-100 in PBS was added to neurons and incubated at RT for 10 min. Primary antibodies incubation using anti-MAP-2 (Synaptic Systems, Göttingen, Germany), and anti-VGLUT1 (Synaptic Systems, Göttingen, Germany) was performed overnight at 4°C. Transduced neurons were incubated with anti-GFP (Abcam, Cambridge, UK) to enhance eGFP fluorescence. Samples were then rinsed five times with PBS and incubated with secondary antibodies conjugated either with 647-Alexa (Thermo Fisher, Waltham, MA, USA), Dylight 549 (Jackson ImmunoResearch, UK) or 488-Alexa (Thermo Fisher, Waltham, MA, USA) for 60 min at RT. After washing in PBS three times, samples were fixed in mounting medium and confocal images were acquired with a Leica TCS SP5 II inverted microscope (Leica Microsystems, Wetzlar, Germany) equipped with a ×63 oil immersion objective. Alexa fluorophores, 488 and 647, were excited with a 488-nm argon laser and with a 633-nm helium-neo laser, respectively. Emission signals were acquired after filtering with 500–550 nm and 640–700 nm bandpass filters, respectively. Dylight 549 was excited with a 543-nm helium-neo laser and emission signal was acquired after filtering with 570–610 nm bandpass filter. Changes in synapse formation were evaluated by analyzing the synaptic contact density per 50 μm length of neuronal structures. Overlapping positive puncta structures were identified between immunolabeled signals generated by presynaptic and postsynaptic markers. Confocal images were analyzed using Fiji ImageJ (Schindelin et al., 2012).

### Electrophysiology

Electrophysiological studies were conducted using the whole-cell voltage-clamp configuration with an EPC-10 amplifier equipped with the PatchMaster software (HEKA, Elektronik). Excitatory postsynaptic currents (EPSCs) were obtained from autaptic neuronal cultures containing a layer of glial cells and a single neuron forming autapses as described (Guzman et al., 2010). The composition of the external recording solution was (in mM): 130 NaCl, 10 NaHCO_3_, 2.4 KCl, 1.25 CaCl_2_, 1.3 MgCl_2_, 10 HEPES, 10 D-glucose, pH 7.3 with NaOH, osmolarity, 310 mOsm. The extracellular solution was supplemented with 20 μM bicuculline (Tocris Bioscience, Bristol, UK) to ensure the recording of EPSCs. Borosilicate patch pipettes with resistances of 3.5–6 MΩ were pulled on a Sutter P-1000 puller (Sutter, Novato, CA, USA) and filled with the intracellular solution containing (in mM): 137.5 K-gluconate, 11 NaCl, 4 MgATP, 0.4 Na_2_GTP, 1.1 EGTA, 11 HEPES, 11 D-glucose, pH 7.3 with CsOH, osmolarity, 310 mOsm. EPSCs were triggered by a brief somatic depolarization from a holding potential of −70 mV to +10 mV during 0.7 ms. Data were registered at 10 or 50 kHz and Bessel filtered at 3.0 kHz. The resulting series resistance was usually less than 12 MΩ, and only neurons with series resistance below 15 MΩ and 70% to 85% resistance compensation were used for analysis. The amplitude of the evoked current was calculated from the baseline amplitude subtracted prior to stimulation artifact. The amount of the RRP of vesicles was measured by application of a hypertonic sucrose solution (500 mM) for 4 s with aid of a fast-flow perfusion system (AutoMate Scientific, Berkeley, CA, USA). The amplitude and charge of evoked EPSCs and the charge of RRP sucrose responses were evaluated by means of the following software: Clampfit (Molecular Devices, San Jose, CA, USA) and OriginPro (OriginLab Corporation, Northampton, MA, USA). The RRP size has been estimated as previously described (Stevens and Sullivan, 1998). The replenishment of RRP was measured after RRP depletion by applying paired-pulses of hypertonic sucrose solution during 3 s at 1 s, 3 s, 7 s, 15 s, 30 s and 60 s. Then, the time course of RRP refilling was analyzed by calculating firstly the percentage of RRP recovery at several interpulse time intervals relative to the last application of hypertonic sucrose (60 s). Second, the percentage of RRP recovery was fitted to a single exponential function to simplify the analysis of data as proposed (Stevens and Wesseling, 1998). To evaluate postsynaptic receptor saturation, 1 mM γ–D-Glutamylglycine (Tocris Bioscience, Bristol, UK) was added to the sucrose solution as described in Supplementary Figure S6. Miniature excitatory spontaneous postsynaptic currents (mEPSCs) were recorded in mass hippocampal neurons at holding potential of −70 mV during 1 min in the presence of 20 μM bicucullin and 1 μM tetrodotoxin (TTX; Biotrend, Köln, Germany) which was added to the standard extracellular solution to avoid spontaneous depolarization of the neurons. Spontaneous events with peak amplitudes higher than 15 pA (~5 times the standard deviation of the background noise) and with charges higher than 25 fC were evaluated using the software MiniAnalysis by Synaptosoft. Data were sampled at 10 kHz and Bessel filtered at 3.0 kHz. Recordings of Ca_V_2.2/Ca_V_β_4b_-mediated currents in HEK293T cells are described in Supplementary Material.

### Statistical Analysis

The data are presented as column scatter dot plots with the mean value ± standard error of the mean (SEM) of each distribution shown by a line. Experiments were conducted in at least three different cell culture preparations with at least three different batches of lentivirus. Statistical significance was analyzed by a comparison between the data sets using one-way analysis of variance (ANOVA). *p* values: *p < 0.05, **p < 0.01, ***p < 0.001 were considered statistically significant. The variability between tested groups was conducted with SigmaPlot version 12.3 (Systat Software, San Jose, CA, USA). The data obtained to calculate the time course of replenishment of RRP were fitted with OriginPro.

## Results

### Ca_v_β Associates With Actin Filaments and the Ca_v_α_1_-Anchoring Domain in a Non-exclusive Manner

**Figure 1:**
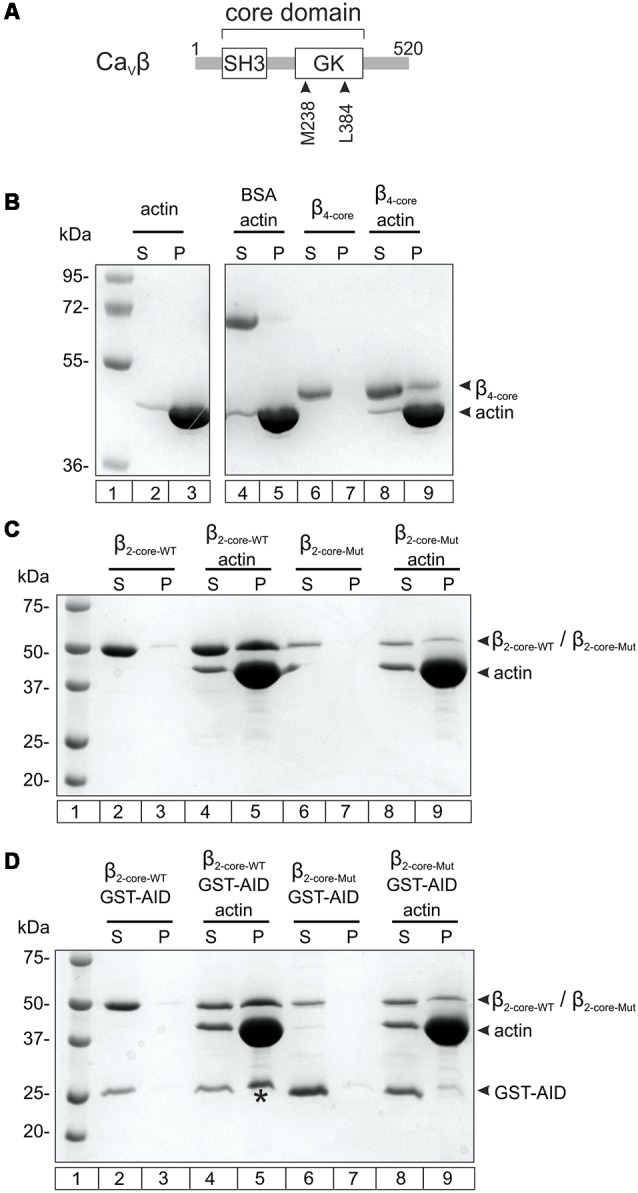
Nonexclusive association between Ca_v_β with the Ca_v_α_1_ anchoring domain and with F-actin. **(A)** Scheme showing the two-domain architecture of Ca_V_β. The numbers correspond to the amino acid position of the Ca_V_β_4b_ sequence used in this study. SH3, Src homology domain, GK, guanylate kinase domain. **(B)** F-actin cosedimentation assay examining the association of Ca_V_β_4b_ core (residues 50–408, β_4-core_) with actin filaments. After incubating actin alone in the polymerization buffer or together with β_4-core_, the proteins were centrifuged and the supernatant (S) and pellet (P) fractions separated and resolved by SDS-denaturing PAGE. The numbers denote the mass in kDa of standard molecular markers (lane 1). The vast majority of actin polymerizes and pelleted after centrifugation (lanes 2–3). BSA, used as negative control, does not co-sediment with F-actin (lanes 4–5). β_4-core_ alone is found in the supernatant after centrifugation (lanes 6–7) and in the pellet fraction when incubated with F-actin (lanes 8–9). For clarity, irrelevant lanes were cropped from the source image (indicated by the white space) and the full image is shown in the Supplementary Figure S1.** (C)** F-actin cosedimentation assay using the wild-type Ca_V_β_2_ core region (β_2-core-WT_) and a mutant with impaired association with the AID site (β_2-core-Mut_). β_2-core-WT_ alone and β_2-core-Mut_ alone are recovered from the supernatant fraction after centrifugation (lanes 2–3 and 6–7, respectively) and in the presence of F-actin they are found in the pellet (lanes 4–5 and 8–9, respectively). **(D)** F-actin cosedimentation assay as in **(C)**, but in the presence of glutathione S-transferase (GST) fused to the AID site (GST-AID). In the absence of F-actin, GST-AID with either β_2-core-WT_ or β_2-core-Mut_ is all found in the supernatant fraction after centrifugation (lanes 2–3 and 6–7, respectively). In the presence of F-actin, GST-AID is recovered in the pellet (denoted by an asterisk) only with β_2-core-WT_ (lanes 4–5), but not with the mutant subunit (lanes 8–9). All assays were repeated at least three times.

We used an *in vitro* co-sedimentation assay to test the association of Ca_V_β_4b_ with actin filaments. For this assay, actin is dissolved in a low salt polymerization buffer to promote the formation of F-actin. After centrifugation, proteins that associate with F-actin are recovered from the pellet fraction. Since recombinant full-length Ca_V_β_s_ are not stable under low salt conditions (Stölting et al., 2015), we purified the core of the human neuronal Ca_V_β_4b_ containing the two highly conserved SH3 and GK domains. We verified that Ca_V_β_4b_ core associates with F-actin as previously described for the core domain derived from Ca_V_β_2_ (Stölting et al., 2015; Figures 1A,B and Supplementary Figure S1).

We next examined if the interaction of Ca_V_β with F-actin and Ca_V_α_1_ are mutually exclusive or not. To assess Ca_V_β-Ca_V_α_1_ association, the highly conserved AID site (Pragnell et al., 1994) was fused to GST (GST-AID) as previously described (Hidalgo et al., 2006). The Ca_V_β_4b_ protein construct bearing a double mutation at two residues critical for association with the AID domain (M238A/L384A, Figure 1A; Chen et al., 2004; Opatowsky et al., 2004) resulted in low yield and poor stability in low salt buffers. Thus, we used the core of Ca_V_β_2_ as background and introduced the two point mutations at the analogous residues. This Ca_V_α_1_ association-deficient Ca_V_β_2_ core mutant is more stable in the F-actin polymerization buffer and preserves the ability to associate with F-actin (Figure 1C). GST-AID co-sedimented together with F-actin in the presence of WT Ca_V_β_2,_ but not of the mutant version with impaired AID association (Figure 1D).

These results demonstrate that GST-AID is mobilized to the pellet fraction, together with F-actin, through its association with Ca_V_β. We conclude that Ca_V_β can simultaneously associate with Ca_V_α_1_ and F-actin.

### Ca_v_β_4b_ Increases the Frequency of Miniature Excitatory Postsynaptic Currents in the Presence of an Intact Actin Cytoskeleton

To study the functional role of the Ca_V_β_4b_/F-actin association in synaptic transmission, we generated two lentiviral gene delivery expression vectors encoding either full-length Ca_V_β_4b_ (hereafter denoted as β_4-WT_) or the Ca_V_β_4b_ M238A/L384A mutant with impaired Ca_V_α_1_-binding (hereafter denoted as β_4-Mut_). Both Ca_V_β_4b_ constructs were linked to either eGFP or mCherry at their carboxy-terminal ends for visualization by fluorescence microscopy. β_4-WT_, but not β_4-Mut,_ expressed in HEK293T associated *in vitro* with GST-AID (Supplementary Figure S2A) and when expressed together with Ca_V_2.2 supported robust ionic currents (Supplementary Figures S2B,C). β_4-Mut_ failed to yield Ca_V_2.2-mediated currents.

**Figure 2:**
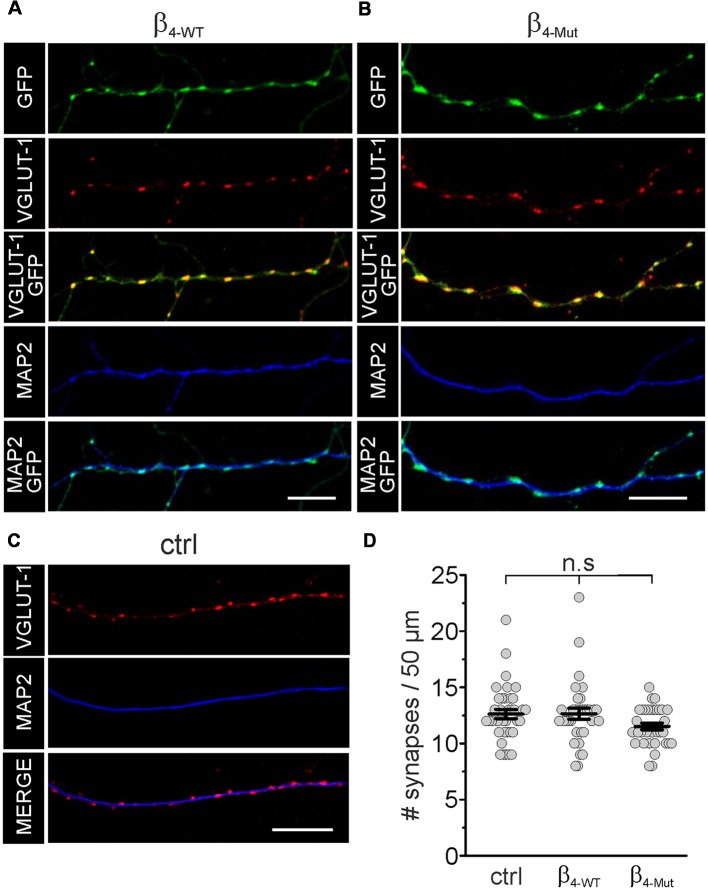
Exogenous WT Ca_V_β_4b_ and the Ca_v_α_1_ association-deficient mutant are targeted to synaptic contacts and do not alter synapse density in hippocampal neurons. Laser scanning confocal images of hippocampal neurons either transduced with Ca_V_β_4b_ WT (β_4-WT_) fused to eGFP **(A)** or with Ca_V_β_4b_ mutant with impaired Ca_V_α_1_ binding (β_4-Mut_) fused to eGFP **(B)** and non-transduced neurons (ctrl; **C**). Neurons were immunolabeled with the glutamatergic presynaptic terminal marker VGLUT-1 (red) and the dendrite-selective marker MAP2 (blue). Ca_V_β_4b_ fluorescence signal is shown in green.** (D)** Scatter dot plot of the number of synaptic contacts per 50 μm dendritic length for the indicated conditions. The number of synapses was quantified by counting the number of VGLUT-1 positive puncta overlapping with Cavβ4-eGFP signal along MAP2-positive neuronal processes per 50 μm length. Lines represent the average value ± standard error of the mean (SEM). n.s, not significant. Scale bar: 10 μm.

In mass cultures of dissociated hippocampal neurons exogenous β_4-WT_ and β_4-Mut_ accumulate in synapses as judged by the co-localization with the presynaptic and postsynaptic markers VGLUT-1 and the MAP2, respectively (Figures 2A,B). We analyzed the number of excitatory synaptic contacts (VGLUT-1 positives puncta) in non-transfected hippocampal neurons and neurons expressing either β_4-WT_ or β_4-Mut_ by immunostaining (Figures 2A–C). Neither β_4-WT_ nor β_4-Mut_ alters synapse density at excitatory synapses (Figure 2D). Thus, exogenous Ca_V_β_4b_ does not modify synaptogenesis.

**Figure 3:**
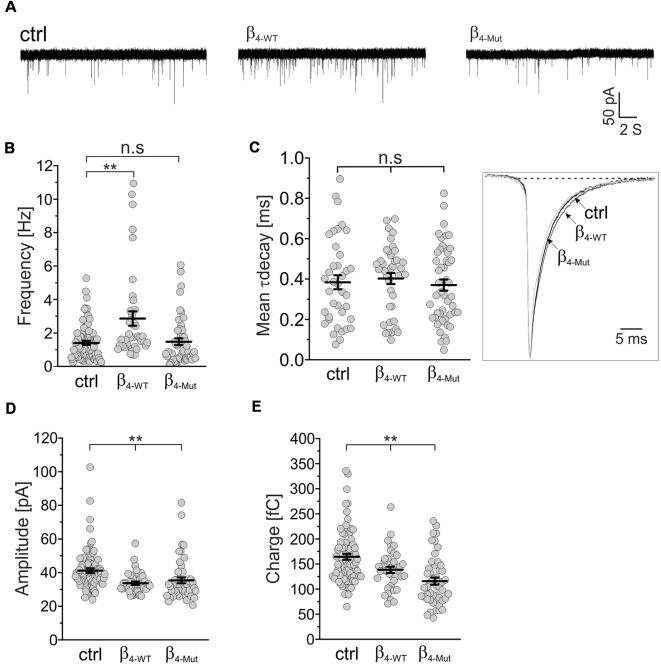
The frequency of excitatory spontaneous release is potentiated only by Ca_v_β_4_ bearing an available Ca_v_α_1_-binding site.** (A)** Representative traces of the miniature excitatory spontaneous postsynaptic currents (mEPSCs) from hippocampal neurons in mass cultures non-transduced (ctrl) or transduced with either β_4-WT_ or the Ca_v_α_1_ association-deficient mutant, β_4-Mut_. **(B)** Statistical analysis of mEPSC frequency in control neurons and neurons transduced with either β_4-WT_ or β_4-Mut_. **(C)** Scatter dot plot of the mean weighted decay time (mean τ decay, left panel) for all the tested conditions as indicated for **(A)** and ensemble averages of the normalized mEPSCs (right panel). **(D)** Scatter dot plot of the mEPSC amplitude recorded from control neurons and neurons overexpressing β_4-WT_ and β_4-Mut_. **(E)** Scatter dot plot of the mEPSC charge transfer estimated for the indicated conditions. Lines represent the average value ± SEM. n.s, not significant; **p < 0.01 one-way ANOVA.

We recorded mEPSCs in mass cultures of hippocampal neurons transduced with the Ca_V_β_4b_-encoding plasmids (Figure 3). We found that β_4-WT_ significantly increased the average frequency of the mEPSCs, whereas β_4-Mut_ produced no changes (Figures 3A,B). No alterations in the mean decay time constant of the averaged mEPSC responses were observed in neurons expressing β_4-WT_ or β_4-Mut_ with respect to non-transduced cells (Figure 3C)_._ The increased mEPSC frequency combined with the absence of alterations in mEPSCs decay time and in synapse density suggests a presynaptic function of Ca_V_β_4b_ in modulating neurotransmitter release as proposed (Wittemann et al., 2000; Xie et al., 2007). Both Ca_V_β_4b_ constructs induced a reduction in the average amplitude and charge transfer of the mEPSCs (Figures 3D,E), resulting in a slight leftward shift of the corresponding cumulative probability curve of the mEPCS with respect to the distribution obtained from non-transduced neurons (Supplementary Figures S3A,B). These changes may arise from a decreased vesicular loading or from a reduction in the density of functional postsynaptic receptors triggered by Ca_V_β_4_. Up-to-date there is no evidence associating Ca_V_β_4_ with SV glutamate loading (Blondeau et al., 2004; Morciano et al., 2005; Takamori et al., 2006). Furthermore, since Ca_V_β_4_ can be also targeted postsynaptically (Wittemann et al., 2000; Xie et al., 2007), we favor the idea that postsynaptic mechanisms account for these rather minor differences.

**Figure 4:**
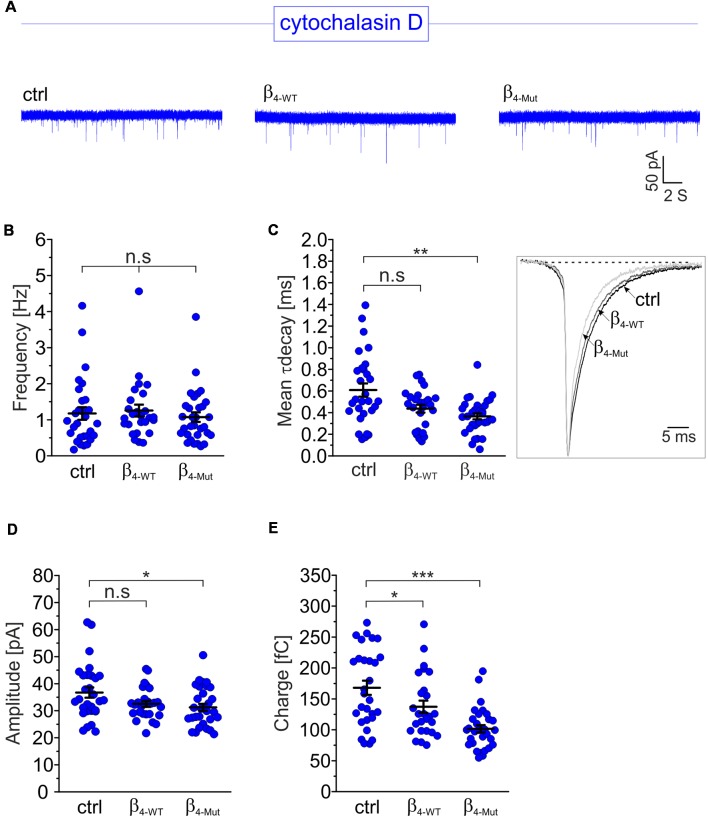
The enhancement of the mEPSC frequency induced by exogenous Ca_v_β_4b_ relies on a competent actin cytoskeleton. **(A)** Representative traces of mEPSC from neurons exposed to the cytochalasin D actin filament disruptor for 1 h (20 μM). For clarity, traces and plots from non-transduced neurons (ctrl) and transduced with either β_4-WT_ or β_4-Mut_ exposed to cytochalasin D are shown in blue in all figures. **(B)** Statistical analysis of mEPSC frequency for neurons exposed to cytochalasin D as shown in **(A)**. **(C)** Scatter dot plot of the mean weighted decay time (mean τ decay, left panel) and ensemble averages of the mEPSCs (right panel) from the neurons exposed to cytochalasin D for the indicated conditions. **(D)** Scatter dot plot of the mEPSC amplitude recorded from the indicated neurons treated with cytochalasin D. **(E)** Scatter dot plot of the mEPSC charge transfer estimated for the indicated conditions. Lines represent the average value ± SEM. n.s, not significant; *p < 0.05, **p < 0.01, ***p < 0.001 one-way ANOVA.

To investigate whether the Ca_V_β_4b_-mediated increase in mEPCS frequency depends on the integrity of the actin cytoskeleton, hippocampal neurons were exposed to the actin filament disruptor cytochalasin D prior to the electrophysiological recordings (Figure 4). Disruption of the actin cytoskeleton fully abolished the increase in the mEPSC frequency induced by β_4-WT_ (Figures 4A,B). Our biochemical analysis indicates that the lack of effect observed for β_4-Mut_ is not due to an impaired F-actin association (Figure 1C). Cytochalasin D treatment did not alter the mEPSC frequency in non-transduced neurons (untreated 1.4 ± 0.12 Hz; cytochalasin D-treated 1.17 ± 0.17 Hz, Figures 4A,B) suggesting that the actin cytoskeleton does not act as a barrier for spontaneous SV fusion (Sankaranarayanan et al., 2003).

The average mEPSCs decay time in non-transduced neurons was significantly increased after exposure to cytochalasin D (from 0.38 ± 0.03 ms to 0.61 ± 0.06 ms in untreated and cytochalasin D-treated neurons, respectively; Figure 4C). This is consistent with the notion that actin filaments participate in the spatial organization of the postsynaptic receptors at synapses (Okamoto et al., 2004). Minor changes in the amplitude and charge transfer of the spontaneous response where observed when comparing untreated neurons with their corresponding cytochalasin D-treated counterparts (Figures 4D,E and Supplementary Figures S3C,D). Nevertheless, a more pronounced effect was found in neurons expressing β_4-Mut_, where the charge transfer decreased from 116 ± 7 fC to 102 ± 6 fC after disruption of the actin cytoskeleton.

Our results show that spontaneous neurotransmitter release is regulated by Ca_V_β_4b_ and that this regulation relies on an intact actin cytoskeleton and competent binding to Ca_V_α_1_.

### A Tripartite Interaction Among Ca_V_β, Ca_V_α_1_ and F-Actin Increases Excitatory Postsynaptic Currents Induced by Depolarization in Hippocampal Neurons

**Figure 5:**
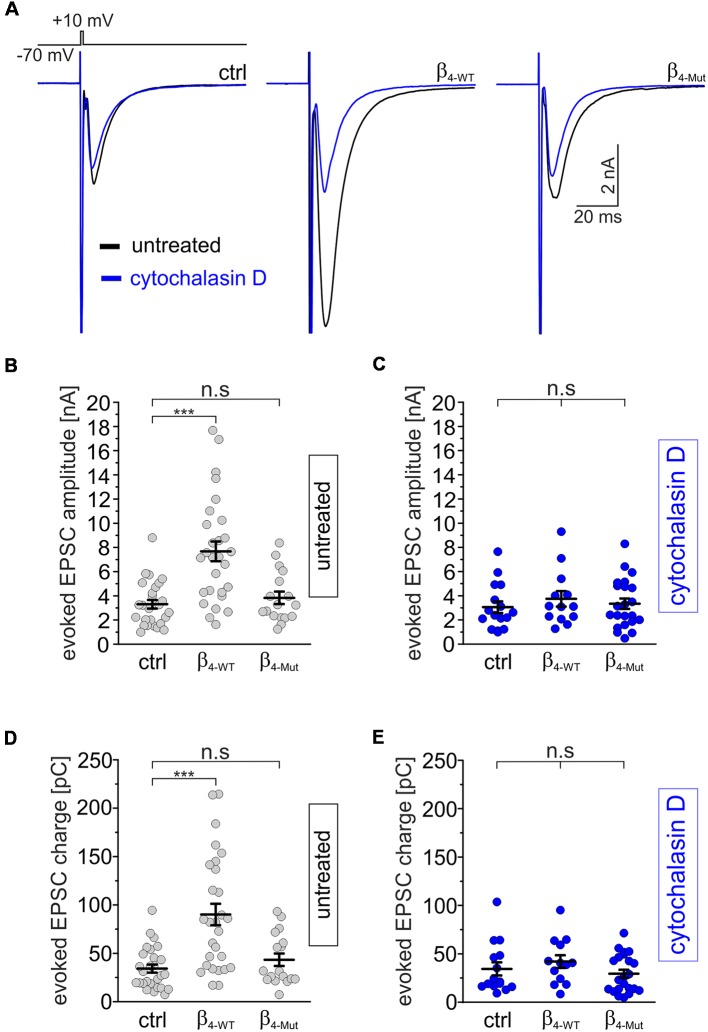
The tripartite interaction among Ca_v_β, Ca_v_α_1_ and F-actin is required for increasing depolarization-evoked EPSCs in individual hippocampal neurons. **(A)** Averaged EPSC response from autaptic hippocampal neurons evoked with the voltage protocol shown at the top. Overlapped average EPSC responses from neurons non-exposed (untreated, black traces) and exposed to 20 μM cytohalasin D for 1 h (blue traces) and expressing either β_4-WT_ or β_4-Mut_ or non-transduced (ctrl) are shown. **(B,C)** Scatter dot plots of the EPSCs amplitude recorded from untreated neurons and cytochalasin D-treated neurons, respectively. **(D,E)** Scatter dot plots of the EPSCs charge transfer estimated from untreated and cytochalasin-D treated neurons, respectively. Lines represent the average value ± SEM. n.s, not significant; ***p < 0.001 one-way ANOVA.

We next recorded evoked EPSCs from autaptic cultures of dissociated hippocampal neurons expressing either β_4-WT_ or β_4-Mut_ (Figure 5). Consistent with previous work (Wittemann et al., 2000; Xie et al., 2007), we observed that exogenous β_4-WT_ resulted in significantly larger amplitudes of the evoked postsynaptic response and increased charge transfer (Figures 5A,B,D). The half-width of the averaged EPSCs were unaffected (Supplementary Figure S4A). Expression of β_4-Mut_ caused no changes in the amplitude, charge transfer and kinetics of the EPSCs (Figures 5A,B,D and Supplementary Figure S4A). Comparison of the EPSCs amplitudes from day 10–14 DIV showed no significant changes suggesting that during this time window, exogenous β_4-Mut_ has reached equilibrium with its endogenous counterparts (data not shown). Moreover, during the same time window, it does affect the RRP size, as shown below. Thus, we do not attribute the lack of effect of β_4-Mut_ to a failure in its ability to actively displace endogenous Ca_V_β_4_.

To assess the impact of the actin cytoskeleton, we exposed hippocampal autaptic cultures expressing either β_4-WT_ or β_4-Mut_ to cytochalasin D before recording the postsynaptic currents. Cytochalasin D treatment did not change the amplitude, charge transfer or kinetics of the evoked EPSC response in non-transduced neurons, but blunted the effect of β_4-WT_ on the EPSC amplitude (Figure 5A, blue traces and **C–E** and Supplementary Figure S4B).

In order to investigate if the effects of cytochalasin D can be mimicked by other actin cytoskeleton disruptor, we treated non-transfected and β_4-WT_-transfected neurons with latrunculin A. We found that this drug also suppressed the increase in EPSC amplitude and charge mediated by β_4-WT_ (Supplementary Figures S5A,C,D).

Altogether, our findings demonstrate that a tripartite interaction among Ca_V_β, Ca_V_α_1_ and F-actin upregulates spontaneous and depolarization-evoked neurotransmitter release *via* activation of presynaptic mechanisms. The requirement of an intact actin cytoskeleton for the increase in synaptic strength regulated by Ca_V_β_4b_ recalls the role of F-actin in mobilizing SVs at different steps of the SV cycle (Cingolani and Goda, 2008).

### Ca_v_β_4_ Increases the Mobilization of Synaptic Vesicles to the Readily Releasable Pool in an F-Actin Dependent Manner

**Figure 6:**
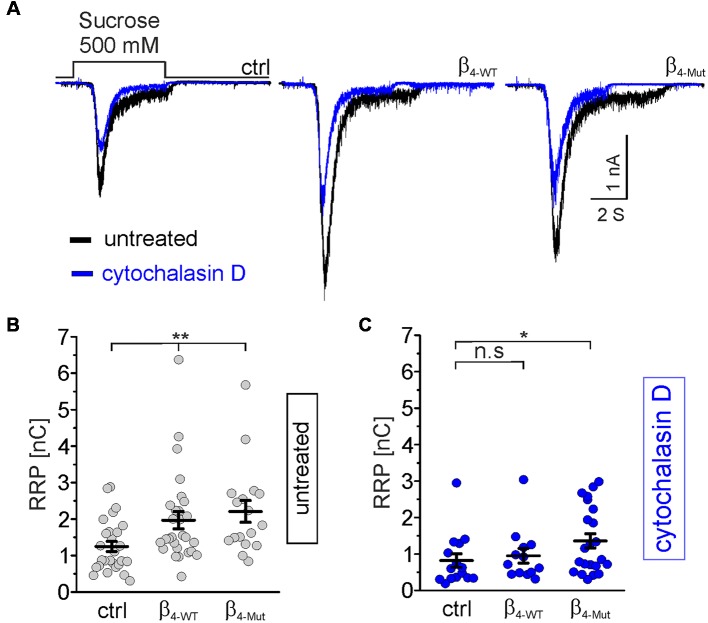
Ca_v_β_4_ WT, as well as the Ca_v_α_1_ association-deficient mutant, increases the readily releasable pool (RRP) of synaptic vesicles (SVs) in an F-actin dependent manner.** (A)** Representative whole-cell responses to hypertonic sucrose solution stimulation (500 mM sucrose) from autaptic non-transduced neurons and either transduced with β_4-WT_ or β_4-Mut_. The responses from untreated neurons and cytochalasin D-treated neurons were overlapped and are shown in black and blue traces, respectively. **(B,C)** Scatter dot plots of the RRP size from untreated and cytochalasin D-treated neurons, respectively. Lines represent the average value ± SEM. n.s, not significant; *p < 0.05, **p < 0.01 one-way ANOVA.

To test whether Ca_V_β/F-actin association is involved in SV translocation, we estimated the size of the RRP. To compare the impact of WT and mutant Ca_V_β_4b_ *per se*, and not due to calcium-dependent effects, we use hypertonic sucrose stimulation that is well-established strategy to measure the RRP size in a calcium-independent manner (Rosenmund and Stevens, 1996). The RRP size was measured 5 s after recording the depolarization-evoked response in the same autaptic neuron (Figure 6). Neurons transfected with β_4-WT_ and β_4-Mut_ displayed an augmented overall RRP size (Figures 6A,B). To assess whether or not our measurements of the RRP size in Ca_V_β_4_-expressing neurons were affected by postsynaptic receptor saturation, we estimated the size of the RRP in neurons expressing β_4-WT_ in the presence and in the absence of 1 mM γ–D-Glutamylglycine, a fast dissociating AMPA receptor antagonist (Liu et al., 1999). The result demonstrated that in our study, postsynaptic receptor saturation does not contribute to the RRP size measurements (Supplementary Figure S6). This is consistent with the previous report using another AMPA receptor antagonist (Schotten et al., 2015). Postsynaptic receptor desensitization contributing to RRP estimations using 500 mM sucrose solution in hippocampal neurons has been negligible (Pyott and Rosenmund, 2002).

In non-transduced neurons, the pharmacological disruption of the actin cytoskeleton led to a reduction in the average of the RRP size (Figure 6A, blue traces, and **C**). Thus, cytochalasin D sensitive actin filaments appear to facilitate, rather than hamper the mobilization of SVs to the RRP under resting conditions.

In neurons transduced with either β_4-WT_ or β_4-Mut_, pharmacological disruption of actin filaments by either cytochalasin D or latrunculin A blunted the increase in the RRP size triggered by this subunit (Figures 6A,C and Supplementary Figures S5B,E). Cytochalasin D-treatment was less efficient in inhibiting the increased RRP size mediated by β_4-Mut_ (Figure 6C). The remaining cytochalasin D-insensitive pool of mobilized SVs may reflect a cluster of remote vesicles that escape the actin network surrounding the active zone.

### The Recovery of the RRP Size After Depletion Is Facilitated by Ca_V_β_4_ Through a Ca_V_α_1_-Dependent Binding and Is Tightly Controlled by F-Actin

**Figure 7:**
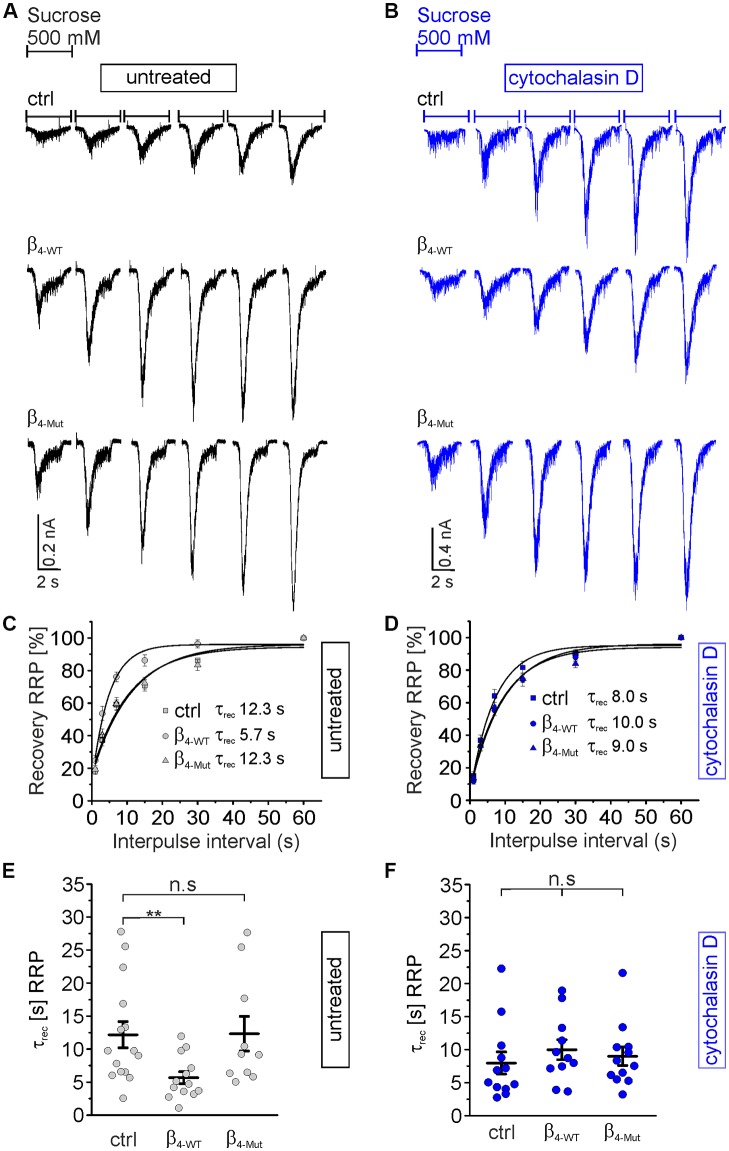
Ca_v_α_1_-binding confers Ca_v_β_4_ the ability to accelerate the time course of recovery of the RRP of SVs after depletion.** (A)** Representative recordings of sucrose responses at increasing time points after depletion from autaptic hippocampal neurons expressing or not the indicated Ca_V_β constructs. **(B)** Representative recordings of sucrose responses as **(A)** but for neurons exposed to cytochalasin D.** (C,D)** Average recovery time course of the RRP replenishment after depletion from data as shown in **(A,B)**, respectively. Continuous lines depict monoexponential fits, and the corresponding time constants (τ_rec_) are reported in the graph.** (E,F)** Scatter dot plot of τ_rec_ from the individual untreated neurons and treated with cytochalasin D, respectively. Lines represent the average value ± SEM. n.s, not significant; **p < 0.01 one-way ANOVA.

Finally, we investigated if the interaction between Ca_V_β_4_ and F-actin also affects the time course of the RRP recovery after depletion (Figure 7). In control autaptic neurons and neurons expressing the Ca_V_α_1_ association-deficient mutant subunit, the RRP is replenished with a time constant of about 12 s (Figures 7A,C,E), which is consistent with the literature (Stevens and Tsujimoto, 1995). However, β_4-WT_ greatly reduces the time constant for the vesicular replenishment of the RRP (τ_rec_ = 5.7 ± 0.9 s as compared to τ_rec_ = 12.3 ± 2.0 s from non-transduced and τ_rec_ = 12.3 ± 2.6 s for neurons transduced with β_4-Mut_). Pharmacological treatment of the neurons with cytochalasin D reverted the effect of the WT subunit and yielded comparable rates of the RRP recovery time among the three groups of neurons, non-transduced and transduced with either β_4-WT_ or β_4-Mut_ (Figures 7B,D,F). These results indicate that the association of Ca_V_β_4b_ with Ca_V_α_1_ and cytochalasin D-sensitive F-actin accelerates the recovery of the RRP after depletion.

## Discussion

We here show that the changes in synaptic transmission elicited by Ca_V_β_4b_ in hippocampal neurons at excitatory synapses are mediated by actin filaments and involve presynaptic mobilization and maturation of SVs toward a fusion-competent state. Direct comparison of the effects of the WT Ca_V_β_4b_ and the Ca_V_α_1_ association-deficient mutant on neurotransmission allowed us to define a novel channel-independent function of this subunit in recruiting vesicles to the RRP.

### The Functional Interplay Among Ca_v_α, Ca_v_β and F-Actin Regulates Synaptic Transmission

Several roles of the actin cytoskeleton at the presynaptic terminal have been considered (Morales et al., 2000; Cingolani and Goda, 2008; Choquet and Triller, 2013; Nelson et al., 2013; Rust and Maritzen, 2015). Actin filaments may serve as tracks for the active translocation of SVs in synaptic terminals or as a scaffold to recruit and promote the interaction of regulatory proteins required for neurotransmitter release. They may also act as a barrier for SV mobilization toward the release site (Halpain, 2003; Sankaranarayanan et al., 2003; Miki et al., 2016).

The increased mean decay time of the miniature response following cytochalasin D-treatment (Figure 4C) is most likely due to the involvement of the actin cytoskeleton on the spatial arrangement of the postsynaptic receptors (Okamoto et al., 2004). Disruption of the actin cytoskeleton with cytochalasin D *per se* did not alter the frequency of the miniature response or the amplitude of the depolarization-evoked EPSC response in non-transfected neurons but prevented their potentiation triggered by exogenous Ca_V_β_4_. Comparable results on evoked response were obtained when actin polymerization was inhibited with latrunculin A (Supplementary Figure S5).

The lack of effect of F-actin disruption on non-transfected hippocampal neurons has been suggested to reflect a counteracting effect between the two opposing presynaptic roles attributed to the actin cytoskeleton; as a barrier as well as a facilitator for the mobilization of SVs (Cingolani and Goda, 2008). Within this framework, our observation that cytochalasin D treatment decreased the RRP size (Figure 6) implies that under our conditions, F-actin acts predominantly as an entryway to repopulate the RRP (Wu et al., 2016).

The tripartite interaction would acquire physiological relevance during high synaptic activity whereby efficient coupling between SVs and Ca_V_α_1_ (and upregulation of the RRP size and their replenishment rate) is required. Enhanced neurotransmission mediated by Ca_V_β/F-actin association would rely on higher availability of this subunit to be rerouted toward this function. In other words, the accessibility of this subunit may be a key determinant for regulating synaptic strength *via* F-actin cytoskeleton and may constitute a rate-limiting step for neurotransmission adaptation in response to the increased activity *via* F-actin association.

Whether spontaneous vesicle release depends on Ca_V_2.x-mediated calcium influx (Kaeser and Regehr, 2014; Williams and Smith, 2018) and whether the same pool of SVs (Groemer and Klingauf, 2007; Ikeda and Bekkers, 2009) or a distinct subset (Fredj and Burrone, 2009) is dedicated to miniature and evoked neurotransmitter release remains under discussion (for review, Truckenbrodt and Rizzoli, 2014). In spite of the controversy, our results demonstrate that the increased frequency of spontaneous release depends on a competent F-actin and intact Ca_V_α_1_ binding site suggesting that close proximity of SVs to Ca_V_α_1_ is required. This requirement may reflect a dependence of the spontaneous release on calcium permeation through Ca_V_2.x channels. In such a case, Ca_V_β/F-actin association tethers SV to the channel complex and promotes spontaneous release upon stochastic channel opening (Ermolyuk et al., 2013). The finding that the same tripartite interaction, Ca_V_β, F-actin and Ca_V_α_1_, is required for the increased evoked response supports the concept of an overlapping pool of vesicles and release sites, which mediates these two modes of neurotransmission.

We propose that the tripartite interaction is mandatory for generating a fusion-competent SV by allowing the coupling of primed SVs with the calcium channel at the release site. Ca_V_β_4_ mutant with no capability to associate with Ca_V_α_1_ (Supplementary Figure S2) fails in tethering the SV to the calcium channel and explains the lack of effect of the mutant Ca_V_β_4_ on the frequency of the spontaneous neurotransmitter release and evoked response.

### Ca_v_β_4_/F-Actin Interaction Recruits Synaptic Vesicles to the Readily Releasable Pool and Is Involved in Its Replenishment

We found that both tested Ca_V_βs—WT and mutant without the ability to associate with Ca_V_α_1_—support the recruitment of SVs to the RRP in concert with F-actin (Figure 6). Thus, the Ca_V_β-regulated increase in the RRP size operates independently of Ca_V_α_1_ function. This is in line with the idea that neurotransmitter release evoked by hypertonic sucrose solution in hippocampal neurons is independent of calcium (Rosenmund and Stevens, 1996). Our results differ from a previous study, which reported unaltered RRP size in hippocampal neurons overexpressing Ca_V_β_4_ and accelerated RRP recovery only after train stimulation. These studies suggested that the latter arose from an increased calcium influx (Xie et al., 2007). We believe that these differences are due to the use of two distinct Ca_V_β_4_ fusion constructs; whereas we linked eGFP to the C-terminus of the β-subunit, the authors of the other study attached the GFP to the N-terminus. The SH3 domain located at the N-terminal moiety of the protein is a main determinant of F-actin binding (Stölting et al., 2015), and N-terminal fusion might have masked functionally relevant protein-protein interactions.

The capability of Ca_V_β_4_ to increase the RRP size hints to a direct role of this subunit in the translocation of vesicles along F-actin (Evans et al., 1998; Cingolani and Goda, 2008; Rust and Maritzen, 2015). This subunit may accumulate in the actin network surrounding SVs at the synaptic terminal and aid their passive recruitment to the RRP or their active trafficking along actin filaments by yet to be established protein-protein interactions. In analogy, Ca_V_β/F-actin association appears to recruit Ca_V_1.2-containing transport vesicles nearby the plasma membrane for recycling in cardiac cells (Stölting et al., 2015; Conrad et al., 2018).

In excitatory hippocampal neurons, SVs within the RRP differ in their readiness to release their content upon a depolarizing stimulus (Hanse and Gustafsson, 2001; Moulder and Mennerick, 2005, 2006; Alabi and Tsien, 2012; Taschenberger et al., 2016; Kaeser and Regehr, 2017). Moreover, reluctant SVs can be converted into fast-releasing ones in an actin-dependent manner by bringing SVs closer to Ca_V_ channels, supporting the so-called positional priming hypothesis (Lee et al., 2012). Within this context, our results are consistent with the notion that Ca_V_β mutant mobilizes a subset of reluctant vesicles to the RRP whereas the WT protein translocate vesicles to the RRP and through its ability to associate with Ca_V_α_1_ renders SV fusion competent.

Diverse protein-protein interactions are involved in positioning SVs and Ca_V_s within nanometer distance at the release site (Eggermann et al., 2011; Davydova et al., 2014; Gundelfinger et al., 2015; Nakamura et al., 2015; Korber and Kuner, 2016; Stanley, 2016; Wang et al., 2016; Kusch et al., 2018; de Jong et al., 2018). Among those, the multi-domain RIM associates directly with Ca_V_2.x channels (Hibino et al., 2002). It has been reported that Ca_V_β also interacts with RIM (Kiyonaka et al., 2007) as well as with synaptotagmin I (Vendel et al., 2006). We here show that Ca_V_β_4_ can simultaneously associate with F-actin and Ca_V_α_1_. This interplay may provide a molecular scaffold to hold in place the plethora of proteins involved in SV docking and priming. Moreover, the dimerization of Ca_V_β (Miranda-Laferte et al., 2011) may also enlarge the number of potential interacting partners regulating synaptic activity. The idea that Ca_V_β may operate as a scaffold between the calcium channel and the release machinery has been anticipated (Vendel et al., 2006; Weiss, 2006; Xie et al., 2007).

β_4-Mut_ preserves its ability to associate with F-actin and to recruit SVs to the RRP but fails to speed up the recovery time of the RRP after depletion. The RRP replenishment was measured following calcium-independent exocytosis so that this lack of effect is not due to its inability to increase calcium current densities. As acceleration of RRP recovery relies on a Ca_V_β_4_ with intact Ca_V_α_1_-binding site, fast replenishment appears to be acquired only after the SV is tethered to Ca_V_α_1_. One can envision that a set of molecular components that prepares the SV and the release site for undergoing fast retrieval is recruited after positioning the SV in close proximity to the calcium channel.

Multiple lines of evidence and theoretical insight agree with the concept that clearance of the release site, rather than SV supply, becomes rate limiting during sustained neurotransmission (Neher, 2010). Regeneration of SVs by the classical clathrin-mediated endocytosis is too slow to account for the effect of Ca_V_β_4_ on the RRP replenishment (Granseth et al., 2007; Granseth and Lagnado, 2008; Yamashita, 2012). Recently, a clathrin-independent ultrafast endocytosis within 50–100 ms after exocytosis was reported in mouse hippocampal neurons (Watanabe et al., 2013, 2014). It occurs at regions flanking the active zone and depends on F-actin and dynamin, two interacting partners of Ca_V_β. Ultrafast endocytosis could primarily serve fast, compensatory retrieval of vesicular membranes and would be followed by the formation of large vesicles that fuse with endosomes and reform a functional SV through a clathrin-dependent process with a much slower time scale in the order of seconds.

Actin appears to mediate virtually all types of SV endocytosis and to facilitate translocation of SVs to the RRP (Wu et al., 2016). Likewise, dynamin is required for the fission of the SV at the cost of GTP hydrolysis and suggested to participate in the clearance of the release sites in different model systems, including hippocampal neurons (Kawasaki et al., 2000; Hosoi et al., 2009; Wu et al., 2009; Soykan et al., 2017). It is recruited to endocytic sites *via* interaction with several proteins containing SH-3 domains. We have previously shown that Ca_V_β interacts with dynamin *via* its SH3 domain and promotes endocytosis (Gonzalez-Gutierrez et al., 2007; Miranda-Laferte et al., 2011). It is tempting to propose that Ca_V_β_4_ facilitates RRP replenishment by promoting dynamin/F-actin-dependent endocytosis that speeds up the removal of SV components and excess of membrane for a new cycle of release at the release site after exocytosis. Since undersupply of SVs and cleared release sites can cause transient synaptic depression during high synaptic activity, upregulation of the RRP size and their replenishment mediated by exogenous Ca_V_β/F-actin association would support stable neurotransmission during sustained synaptic activity.

In conclusion, a physical association between Ca_V_β_4_, Ca_V_α_1_ and F-actin appears to be a sine-qua-non condition for bringing the SV within the permissive range of the Ca_V_ calcium nanodomain for release (Naraghi and Neher, 1997; Neher and Sakaba, 2008; Park et al., 2012; Nakamura et al., 2015; Stanley, 2015, 2016). This scenario places Ca_V_β_4_ as essential for the maturation of the SVs toward a fusion-competent state acting as a tether for the functional priming of the SV during spontaneous and depolarization-evoked synaptic transmission.

## Ethics Statement

This study was carried out in accordance with the recommendations of the German Law for the Protection of Animals. The protocol was approved by the Forschungszentrum Jülich GmbH and LANUV (State Agency for Nature, Environment and Consumer Protection) of North Rhine-Westphalia.

## Author Contributions

GG designed and performed the experiments, analyzed the data and edited the manuscript. RG analyzed data and edited manuscript. NJ produced all recombinant proteins and performed the biochemical assays and immunostainings. PH designed and supervised the research, and wrote the article.

## Conflict of Interest Statement

The authors declare that the research was conducted in the absence of any commercial or financial relationships that could be construed as a potential conflict of interest.
