# Supplementary material for: A Tripartite Interaction Among the Calcium Channel α1- and β-Subunits and F-Actin Increases the Readily Releasable Pool of Vesicles and Its Recovery After Depletion
Source: Front Cell Neurosci. 2019 May 3;13:125. doi: 10.3389/fncel.2019.00125 (PMC6509170; doi:10.3389/fncel.2019.00125)
Supplement: Supplementary file 1 [file Data_Sheet_1.pdf]

Supplementary Material

A tripartite interaction among the calcium channel α_1- and β-subunits and F-actin increases the readily releasable pool of vesicles and its recovery after depletion

Gustavo A. Guzman, Raul E. Guzman, Nadine Jordan, Patricia Hidalgo*

*** Correspondence:** pa.hidalgo@fz-juelich.de

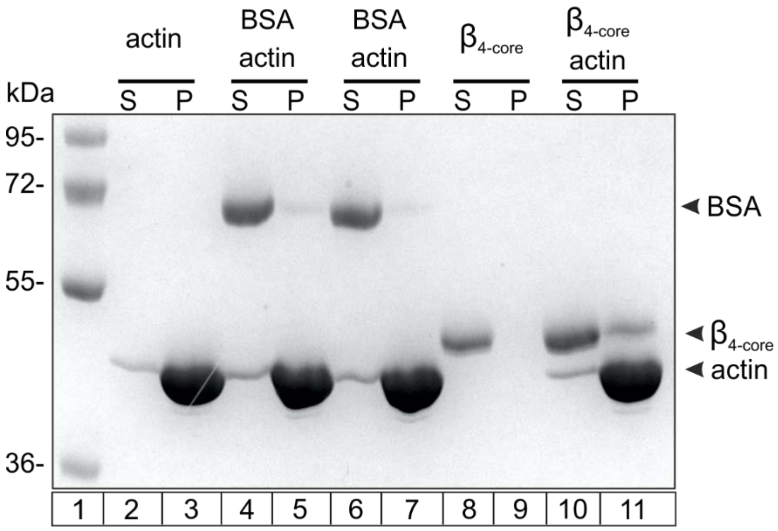

Supplementary Figure 1. **Source image of the F-actin cosedimentation assay examining association of wild-type Cavβ4b core (residues 50-40, β4-core) shown in the main text.** Lanes 4-5 and 6-7 correspond to replicas of the negative control assay using two different sources of BSA. The gel shown in Figure 1B was cropped to remove lanes 4-5. F-actin polymerization buffer contained: 10 mM Tris-HCl, 0.2 mM $CaCl_2$, 50 mM KCl, 2 mM $MgCl_2$, 1mM ATP, pH 8.0.

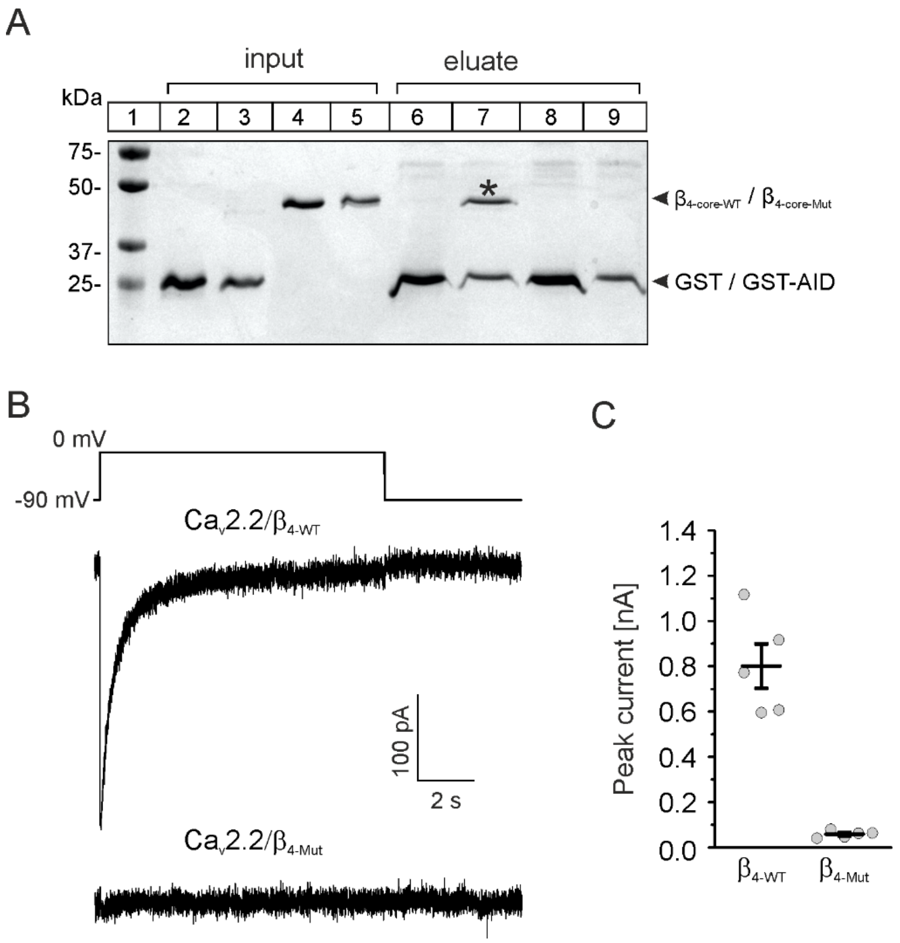

Supplementary Figure 2. **Wild-type Ca$_V\beta_{4b}$, but not the M238A/L384A mutant, binds in vitro to the Ca$_V\alpha_1$-anchoring domain and facilitates Ca$_V$2.2-mediated currents. (A)** In vitro binding assay of wild-type Ca$_V\beta_{4b}$ core domain (residues 50 to 408, $\beta_{4\text{-core-WT}}$) and bearing the amino acid substitutions M238A/L384A ($\beta_{4\text{-core-Mut}}$). $\beta_{4\text{-core}}$ constructs were expressed in *E.coli* and purified from the crude lysate by metal affinity followed by size-exclusion chromatography. GST fused to the Ca$_V\alpha_1$-anchoring domain, AID, (GST-AID) or GST alone, used as negative control, were immobilized on glutathione-sepharose beads and used as bait in an standard GST pull down assay. $\beta_{4\text{-core}}$ constructs were incubated the coupled beads and after several washing steps, bound proteins were eluted with SDS-loading buffer and resolved in SDS denaturing PAGE. $\beta_{4\text{-core-WT}}$ (marked with an asterisk) pulled down with GST-AID but not with GST alone, whereas neither GST-AID nor GST pulled down $\beta_{4\text{-core-Mut}}$. Reaction buffer contained: 50 mM Tris-HCl, 300 mM NaCl, 0.1% Triton X-100, 1 mM EDTA, pH 8.0. **(B)** Representative current traces mediated by heterologous Ca$_V$2.2 coexpressed in HEK293T with the full length Ca$_V\beta_{4b}$ constructs used in this study for transducing hippocampal neurons. For visualization of transfected cells, Ca$_V$2.2 was fused to GFP while Ca$_V\beta_4$ constructs to m-Cherry. Currents from cells coexpressing Ca$_V$2.2 with either $\beta_{4\text{-WT}}$ or $\beta_{4\text{-Mut}}$ are shown in the upper and lower panel, respectively and were elicited by a 10 s voltage step

to 0 mV from a holding potential of -90 mV and using Ba^{2+} as charge carrier. Borosilicate pipettes with resistances of 0.9-2 MΩ were used. Series resistance compensation led to a voltage error of less than 5 mV. The external recording solution contained (in mM): 140 TEA-$MeSO_3$, 10 $BaCl_2$, 10 HEPES, pH 7.3 adjusted with TEA-OH while the internal solution contained (in mM): 135 Cs-$MeSO_3$, 10 EGTA, 5 $CsCl_2$, 1 $MgCl_2$, 4 MgATP, 0.4 Na_2GTP, 10 HEPES, the pH was adjusted to 7.3 with CsOH. The data analysis was performed using a combination of FitMaster (HEKA, Lambrecht, Germany), OriginPro (OriginLab Corporation, Northampton, MA, USA) and Excel (Microsoft) software. All recordings were performed at room temperature. **(C)** Scatter dot plot of the peak current from the cells expressing either $Ca_V2.2/\beta_{4\text{-WT}}$ or $Ca_V2.2/\beta_{4\text{-Mut}}$ channel complexes. Lines represent the average value ± S.E.M.

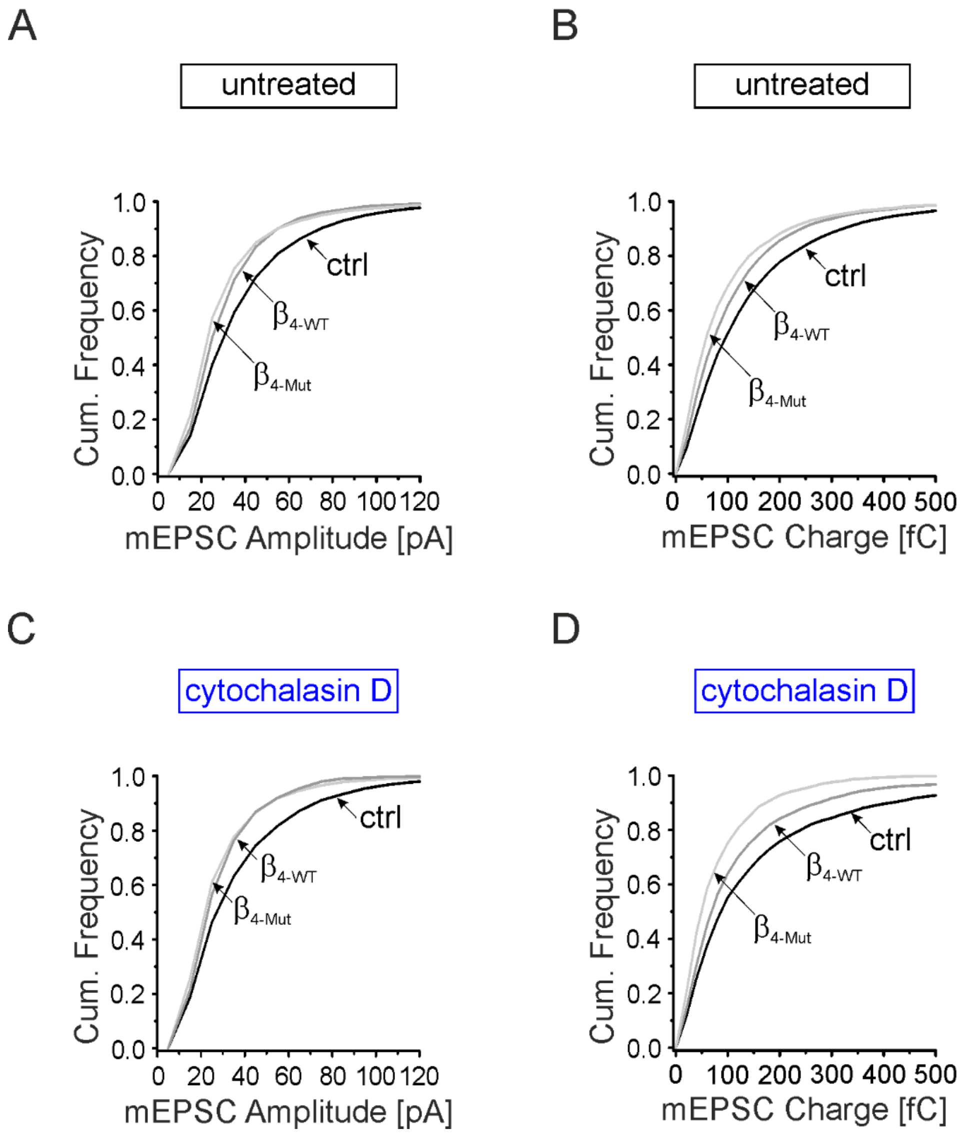

Supplementary Figure 3. **Comparison of the cumulative frequency distributions of the mEPSC amplitude and charge transfer from untreated hippocampal neurons and treated with cytochalasin D.** Cumulative amplitude frequency distribution **(A)** and cumulative charge transfer frequency distribution **(B)** for untreated neurons expressing either $\beta_{4\text{-WT}}$ or $\beta_{4\text{-Mut}}$ and non-transduced neurons (ctrl). Cumulative amplitude **(C)** and charge transfer **(D)** frequency distributions for non-transduced and transduced neurons treated with cytochalasin D.

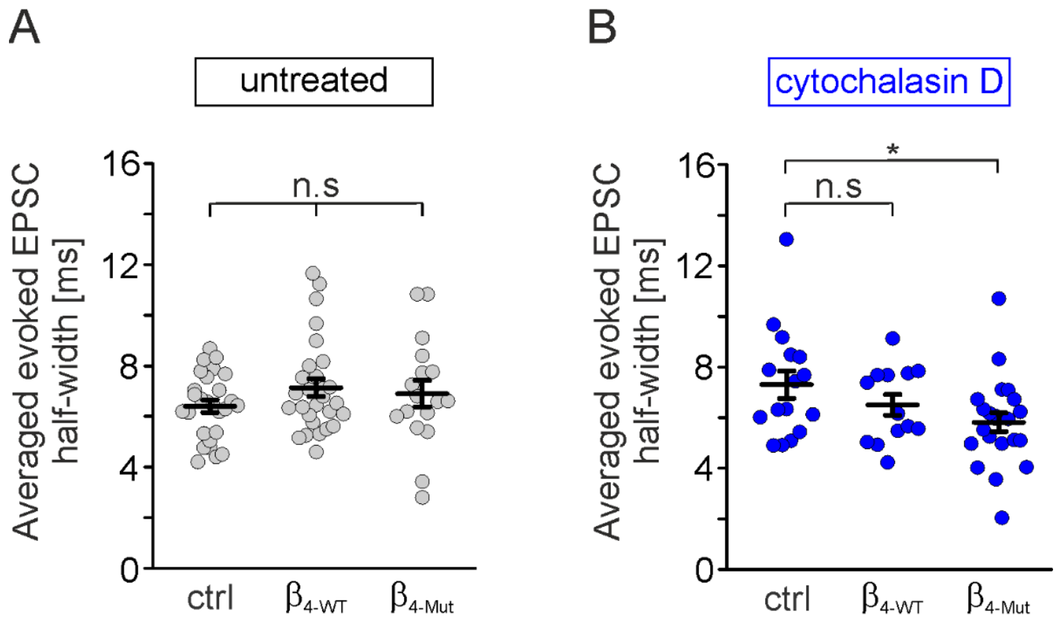

Supplementary Figure 4. **Comparison of the half-widths of the averaged depolarization-evoked EPSC response from neurons treated or not with cytochalasin D.** Scatter dot plot of the half-widths of the averaged evoked EPSC from untreated neurons **(A)** and neurons exposed to cytochalasin D **(B)** and expressing or not the indicated constructs. Lines represent the average value ± S.E.M. n.s, not significant; *$p < 0.05$ one-way ANOVA.

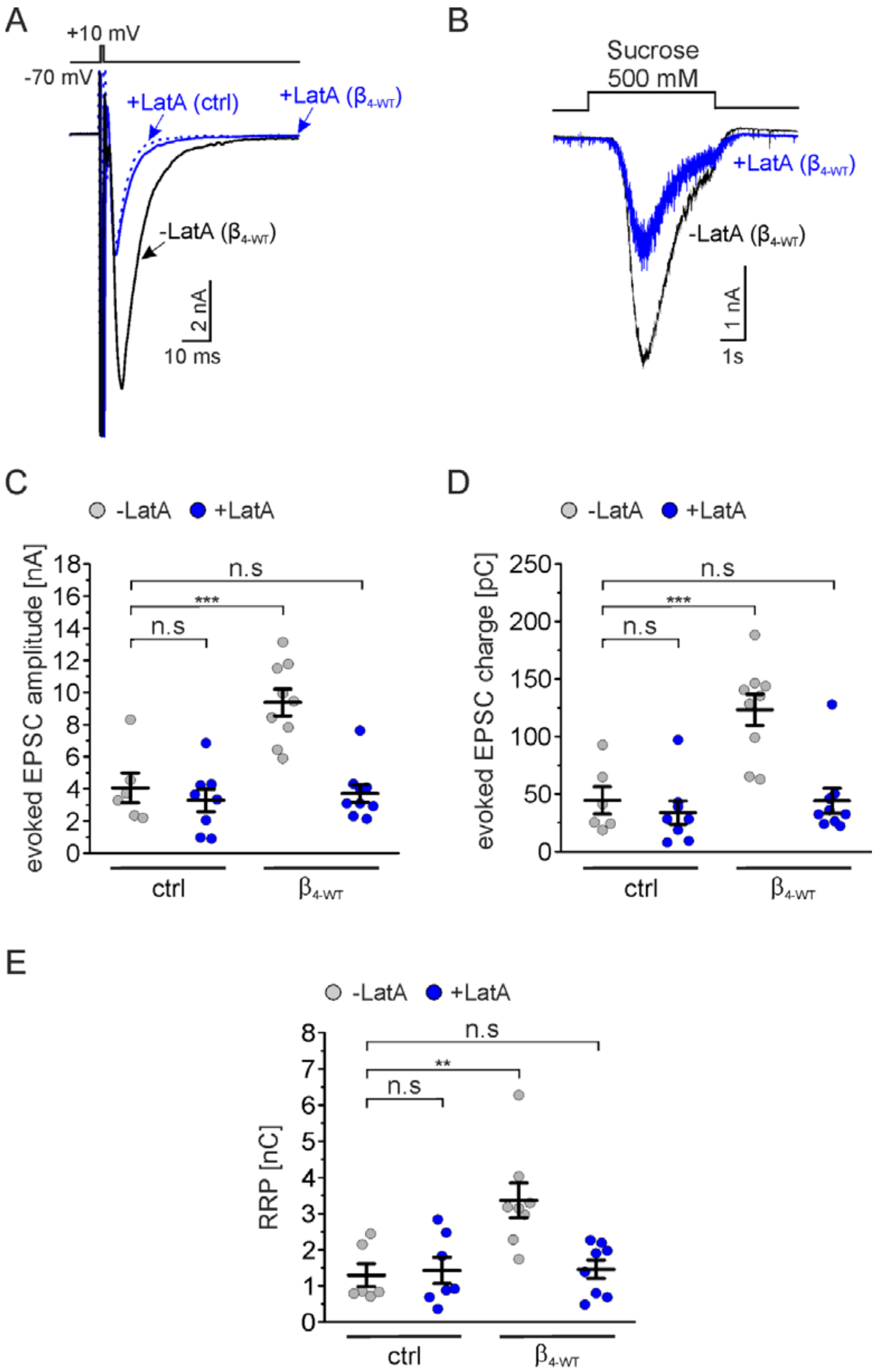

Supplementary Figure 5. Disruption of the actin cytoskeleton by latrunculin A inhibited the Cavβ_4-mediated increase in the excitatory postsynaptic currents and the RRP size. (A,B)

Representative EPSCs from autaptic hippocampal neurons evoked with the voltage protocol, shown at the top, or with 500 mM hypertonic sucrose solution. Overlaying current traces from non-transfected neurons exposed for 10 minutes to 20 µM latrunculin A (+LatA, blue dotted trace) and from neurons expressing $\beta_{4\text{-WT}}$ either treated (+LatA ($\beta_{4\text{-WT}}$), blue trace) or not treated with latrunculin A (-LatA ($\beta_{4\text{-WT}}$), black trace). For clarity we omitted the sucrose response for non-transfected neurons exposed to latrunculin that fully overlaps with the trace from +LatA ($\beta_{4\text{-WT}}$). **(C-D)** Scatter dot plots of the depolarization-evoked EPSCs amplitude and charge transfer estimated from untreated neurons and treated with latrunculin A, as indicated. **(E)**. Scatter dot plot of the readily releasable pool size (RRP) from neurons under the indicated conditions. Lines represent the average value ± S.E.M. n.s, not significant; ** $p < 0.01$, *** $p < 0.001$ one-way ANOVA.

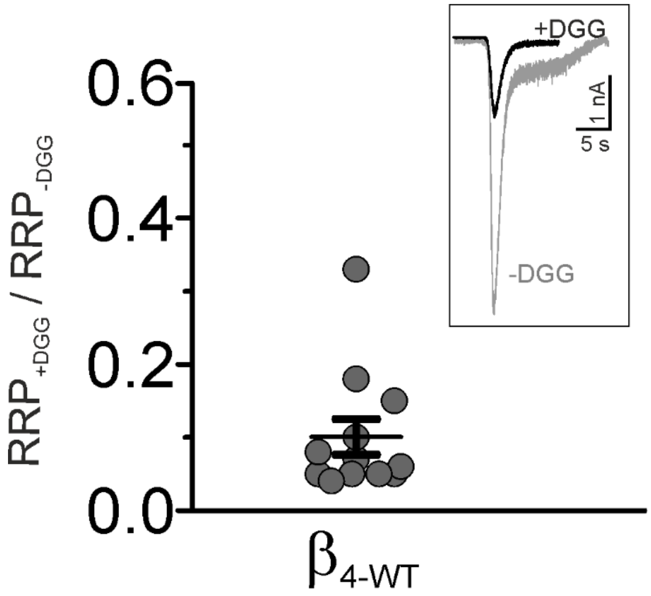

Supplementary Figure 6. **Effect of the fast dissociating AMPA receptor antagonist γ–D-Glutamylglycine (DGG) on the RRP size estimated from neurons overexpressing Ca$_V$β$_4$-wild-type.** Scatter dot plot of the fraction of readily releasable pool size (RRP) estimated after DGG application (n=12). The inset shows representative sucrose responses (500 mM) from autaptic hippocampal neurons expressing β$_{4\text{-WT}}$ in the presence (black trace) or absence (gray trace) of 1mM DGG. After the first sucrose application for 5 s in the absence of DGG, the neuron was perfused with external solution for 30 s followed by 30 s with ringer containing 1mM DGG prior to the application of second sucrose solution containing this time 1mM DDG for 5 s. Lines represent the average value ± S.E.M. The result indicates that postsynaptic receptor saturation does not contribute to the RRP size measurements from neurons expressing Ca$_V$β$_4$ using hypertonic sucrose stimulation (Liu et al., 1999).
